# Can Chronic Remote Cortical Hypoperfusion Induced by Thalamic Infarction Cause Damage of Tracts Passing through Those Hypoperfused Regions?

**DOI:** 10.3389/fneur.2013.00156

**Published:** 2013-10-07

**Authors:** Eloi Magnin, Ludivine Chamard, Fabrice Vuillier, Laurent Tatu, Eric Berger

**Affiliations:** ^1^Department of Neurology, Memory Center (CMRR), Besançon University Hospital, Besançon, France; ^2^Laboratory of Anatomy, University of Franche-Comté, Besançon, France

**Keywords:** thalamus, diaschisis, vascular leukoencephalopathy, SPECT, hypoperfusion

## Abstract

We report the case of a woman presenting with changes on cerebral imaging a year and a half after a bi-thalamic (predominantly left-sided) infarction including lateral and medial thalamic nuclei. Lateral geniculate body and pulvinar were not damaged. Hypoperfusion was observed in left cortical and basal structures. White matter FLAIR hyperintense lesions occurred in the left hemisphere and the occipital region 1 year and half after stroke. Medial and lateral thalamic nuclei are not highly connected to the occipital cortex. Therefore, in addition to Wallerian degeneration after thalamic stroke, we hypothesize that the chronic left temporal hypoperfusion induced by diaschisis can lead to a lateralized chronic hypoxic damage of the occipital fiber tract (optic radiation) that passes through the temporal lobe.

## Introduction

Remote hypoperfusion in stroke is frequently considered as a diaschisis induced by deactivation of regions highly connected with the damaged structure (1). Wallerian degeneration, corresponding to axonal orthograde degeneration after the damage inducing white matter abnormalities, might participate in this pathophysiological process (2). Remote hypoperfusion, especially crossed cerebellar hypoperfusion, is associated with poor recovery prognosis (3, 4). However, in addition to the functional modification, Cobalt-55 PET suggests that damage occurs in the regions where cerebral blood flow is decreased (5).

We report the case of delayed unilateral left leukoencephalopathy in chronic hemispheric hypoperfusion after a left medial and lateral thalamic infarct. As the leukopathy was not only restricted in the white matter highly connected to medial and lateral thalamic nuclei (i.e., frontal and temporal lobes), but also in the left occipital white matter, a potential toxicity of the chronic remote hypoperfusion is discussed.

## Case Report

We report the case of an 81-year-old woman presenting with changes on cerebral imaging a year and a half after a bi-thalamic (predominantly left-sided) infarction including lateral and medial thalamic nuclei.

**Figure 1:**
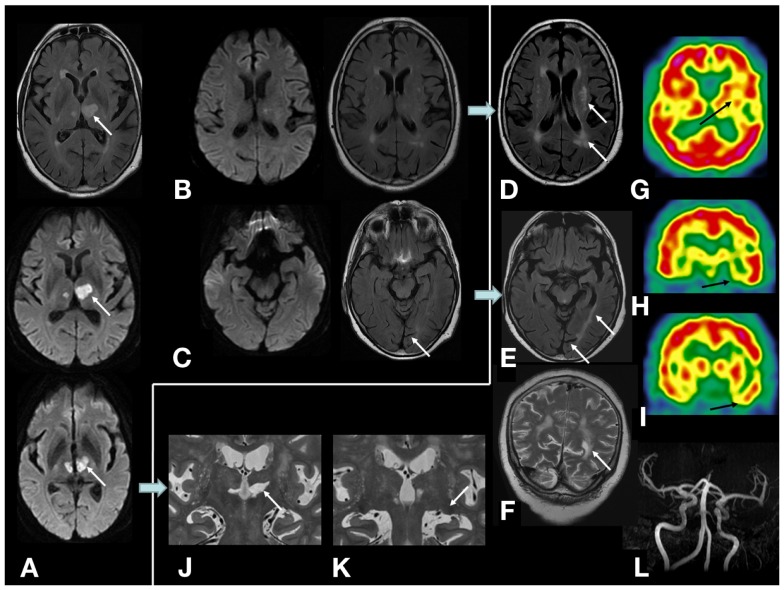
**On MRI examination, initial diffusion-weighted, and FLAIR sequences showing (A) initial bi-thalamic infarction predominantly on the left side; (B,C) no lesion of optic radiations, temporal, and parietal lobes was observed in the acute phase of stroke; (D–F) 1 year and 8 months later, left white matter hyperintense lesion appeared in the left external capsule and semiovale center and temporo-parieto-occipital white matter, cortical and subcortical atrophy on the left occipital lobe (calcarine sulcus)**. No occipital or parietal lesion occurred; **(G–I)** cerebral perfusion SPECT (^99^Tc-HMPAO) showed hypoperfusion in the left striatum, thalamus, perisylvian, and temporal cortex; **(J,K)** lesion of left medial and lateral thalamic nuclei. Lateral geniculate body and pulvinar were not damaged; **(L)** no intracranial stenosis were found on time-of-flight MR angiography.

In the acute phase, she presented isolated language disorders (NIHSS: 1). No hemianopsia or quadranopsia was observed. Initial MRI examination showed bi-thalamic infarction predominantly on the left side including medial and lateral thalamic nuclei. Lateral geniculate bodies, pulvinar, optic radiation, temporal, and parietal lobes were not damaged (Figure 1). No hypertension or other vascular risk factors (diabetes and thyroidopathy, hypercholesterolemia) except age were found. Color duplex sonography found no carotid stenosis. Time-of-flight MR angiography found no intracranial stenosis. About 1 year and 8 months later, white matter FLAIR hyperintense lesions and atrophy were observed in the left hemisphere and the occipital region. Hypoperfusion was observed in left cortical and basal structures (Figure 1). Fundus of eye and renal function were normal. No headache was reported. No systemic inflammation, blood count abnormality, and autoimmune antibody were found. No lumbar puncture was available. Despite these changes shown on neuroimaging, neuropsychological performances showed the same level of thalamic aphasia and executive dysfunctions as the previous year. No visual field defect occurred during the follow-up. The hypothesis of neurodegenerative pathology was thus eliminated.

## Discussion

Changes in white matter after thalamic lesions are reported with DTI sequences in an amnesic patient (2). Hypometabolism is considered as diaschisis and changes in white matter are considered as Wallerian degeneration of the memory network. However, their results are based on regions of interest and also showed a diffuse decreased value of fractional anisotropy in the left hemisphere. De Reuck et al. suggest that damage occurred in the regions deactivated by diaschisis (5). Left FLAIR hyperintense modifications in our case were homolateral to the larger and most symptomatic thalamic infarction without intra or extracranial stenosis. No such an accumulation was observed on the right hemisphere suggesting that the abnormalities observed were not due to conventional chronic vascular leukopathy which is usually bilateral and fairly symmetric. The apparent clinical stability of the patient, biological and imaging results make a non-atherogenic process, as vasculitis for example, unlikely, despite absence of arteriography, CSF analysis, brain and leptomeningeal biopsy. Left abnormalities on FLAIR were not restricted to the language and executive network, and encompassed occipital white matter including optic radiation. No DTI sequence was available; however Johansen-Berg et al. showed that medial and lateral thalamic nuclei are not highly connected to the occipital cortex (6). Wallerian degeneration along fibers connecting the thalamus with cortical and basal structures cannot explain the occipital white matter abnormalities and cortical and subcortical atrophy. Therefore, in addition to Wallerian degeneration induced by the infraction, we hypothesize that the chronic left temporal hypoperfusion induced by diaschisis can lead to a lateralized presymptomatic chronic hypoxic damage of the occipital fiber tract that passes through the temporal lobe (i.e., optic radiation) and then a Wallerian degeneration of the occipital cortex.

## Concluding Remarks

Further studies are required to confirm the direct toxicity of the “functional” hypoperfusion. If it is confirmed, it may be an aggravating factor playing a role in the poor prognosis of post-stroke chronic diaschisis (4) and should be considered as a potential therapeutic target for brain stimulation or vasoactive treatment during rehabilitation therapy.

## Authors Contribution

Eloi Magnin: drafting/revising the manuscript for content, including medical writing for content; analysis of interpretation of data; acquisition of data. Ludivine Chamard: acquisition of data. Eric Berger: acquisition of data. Laurent Tatu: analysis of interpretation of data. Fabrice Vuiller: acquisition of data; analysis of interpretation of data.

## Conflict of Interest Statement

The authors declare that the research was conducted in the absence of any commercial or financial relationships that could be construed as a potential conflict of interest.
